# Dynamic Interactions between the Gut Microbiome, Health, and Metabolic Disorders in Preterm Infants

**DOI:** 10.4014/jmb.2508.08033

**Published:** 2025-10-15

**Authors:** Ki Beom Jang, Eunsol Seo, Younghoon Kim

**Affiliations:** Department of Agricultural Biotechnology and Research Institute of Agriculture and Life Science, Seoul National University, Seoul 08826, Republic of Korea

**Keywords:** Preterm birth, gut microbiota, early development, intestinal health, inflammation, probiotics

## Abstract

Preterm birth, which occurs before the entire gestation is completed, causes serious health challenges for newborns. The gastrointestinal tract often shows delayed development and poor function in nutrient utilization and immune response. Preterm neonates are faced with difficulties in growth, are more vulnerable to infections, and have a higher risk of developing inflammatory diseases. One key factor in these outcomes is the abnormal development of intestinal microbiota. In premature neonates, gut microbes tend to show lower diversity and changes in several bacteria. The gut dysbiosis can disrupt gut function and contribute to complications like necrotizing enterocolitis, sepsis, and even long-term metabolic and neurodevelopmental disorders. This review brings together current research on how premature birth affects the growth, gut development, and overall health of infants, with a focus on changes in microbial colonization. In addition, we discuss how gut microbiota plays a role in shaping the immune system and influencing brain development. There is growing interest in using breast milk, specialized nutrition, and probiotics to improve microbial balance in premature infants. Preterm birth alters intestinal development and microbial colonization, making infants vulnerable to gut dysbiosis and disease. These changes contribute to a heightened risk of neonatal diseases such as NEC, sepsis, and long-term neurodevelopmental and metabolic disorders. Evidence highlights the critical role of commensal microbes and their metabolites in promoting epithelial maturation, immune balance, and gut–brain signaling. Advancing neonatal care will require precision approaches based on microbial profiling, targeted nutritional strategies, and a deeper understanding of host–microbe interactions to improve health in preterm infants. Therefore, this review focuses on integrating recent evidence of microbial development and preterm infant health, highlighting the gut–brain axis in relation to metabolic disorders, and emphasizing how gut dysbiosis links clinical outcomes with mechanistic insights in neonatal care.

## Introduction

Prematurity refers to the birth of an animal before the completion of the gestation period. Preterm birth in humans, defined as delivery before 37 weeks of gestation, affects approximately 13.4 million infants annually, approximately 10% of global births according to the World Health Organization [1]. Preterm birth also occurs in livestock production, due to low survival rates and the lack of a standard framework for preterm livestock birth [2]. Preterm birth is not commonly recognized as a distinct category of concern in animal production, and it is mainly highlighted as an animal model for human research. However, preterm birth can occur and negatively affect production by increasing mortality, compromising growth, and raising disease susceptibility [3]. Preterm birth is also recognized as a critical challenge, contributing to various health issues with a higher risk of extra-uterine growth retardation, neurodevelopmental impairment, and metabolic diseases later in life [4]. These adverse outcomes are closely related to the insufficient development of internal organ systems, including the gastrointestinal, respiratory, and immune systems, during the gestational period [5]. Unfortunately, the cause of preterm birth is not clear due to multiple mechanisms of various biological and environmental factors, such as uteroplacental health, maternal condition, viral and bacterial infection, chronic stress, or other immunological responses [6].

The intestine is a major organ of the digestive system that is exposed to the external environment, playing a central role in nutrient digestion and absorption, and maintaining systemic homeostasis through its mucosal immune function [7]. However, preterm birth leads to gastrointestinal immaturity, resulting in impaired nutrient utilization and intestinal barrier function [8]. These vulnerabilities, combined with distinct patterns of microbial colonization, are compared with those of term infants. Such alterations increase susceptibility to neonatal conditions, including sepsis-associated enteropathy and necrotizing enterocolitis (NEC) in the neonatal period, and may also contribute to an increased risk of developing chronic disorders like inflammatory bowel disease (IBD) later in life [9]. The development of intestinal microbiota can also be affected by clinical management practices associated with preterm birth, including delivery method, feeding practices, or early exposure to medication [10, 11]. Extensive research, conducted in humans, animal models, and *in vitro* systems, has highlighted the pivotal role of the intestinal microbiota in host physiology [12-14]. In preterm infants, the development of the intestinal microbiota is often altered and suboptimal, typically characterized by delayed colonization, reduced diversity, and gut dysbiosis compared to term infants [15, 16]. Considering the significant impact of early-life microbiota on host development, it is critical to understand how microbial colonization is shaped by prematurity. This review aims to summarize current knowledge on the development of the intestinal microbiota in preterm neonates and examine the implications of microbial development for health and disease.

## Effects of Prematurity on Growth and Health

### Body Composition Development

Preterm neonates typically have a higher proportion of body fat mass than term neonates, which is negatively correlated with gestational age and positively associated with body weight increase [17, 18]. According to a meta-analysis conducted by Johnson *et al*. [19], preterm infants had a 3% greater total body fat and much less fat-free mass, about 460 g in the body, compared to full-term infants. These changes may increase the risk of developing metabolic syndrome and cardiovascular diseases in the future [20]. Viswanathan *et al*. [21] demonstrated that maintaining a healthy balance between fat-free mass and fat mass is crucial for neurodevelopment and metabolic health in preterm infants. Based on previous findings, preterm infants have different body compositions, which can be explained by several reasons. Firstly, immature metabolic systems and challenges hinder effective nutrient utilization, affecting growth and body composition [22, 23]. The immature gastrointestinal system in preterm infants can lead to malabsorption and feeding difficulties, further impacting their nutrient intake and utilization [24]. In addition, hormonal secretion imbalances play a significant role in the altered body composition of preterm infants [22, 25]. One of the causes suggested for the impacts of prematurity is the extreme environmental stress [26] after birth, leading to increased circulating cortisol levels, which in turn affect leptin metabolism and the hypothalamic-pituitary-adrenal (HPA) axis [27]. The endocrine system, which regulates body metabolism, would not be fully developed in preterm infants. Key metabolic hormones may not function optimally, impairing nutrient metabolism [28]. The hormonal dysregulation can result in increased fat deposition and altered muscle development [28]. Understanding these factors is crucial for developing effective nutritional and medical strategies to support the growth and development of preterm infants [29, 30].

### Impact of Prematurity on Physiological and Metabolic Development

Prematurity can adversely affect several organ systems, including the neurological, respiratory, and cardiovascular systems (Table 1). During the neonatal period, brain growth and maintenance require a continuous energy supply. However, providing adequate nutrition to premature infants is challenging due to immature gastrointestinal function. Total parenteral nutrition (TPN) is commonly used but is limited in the amount and duration it can be safely administered. Long-term TPN use may impair intestinal development and induce metabolic liver dysfunction [31]. Therefore, early enteral feeding is initiated, and TPN is gradually reduced while carefully introducing digestive feeding [32]. Under nutrient-deficient conditions, tissue and protein breakdown provide alternative energy sources through gluconeogenesis from amino acids or ketogenesis for energy needs [33].

The blood system plays a central role in supporting growth and development by transporting nutrients and oxygen, removing waste products via the liver and kidneys, contributing to systemic immune defense, and facilitating adaptation to environmental changes. Preterm birth is also associated with an increased risk of cardiovascular diseases, including cardiometabolic and cardiopulmonary morbidities later in life [34, 35]. Adults born preterm often exhibit alterations in cardiac structure and function, such as smaller ventricular volumes, increased ventricular mass, and both systolic and diastolic dysfunction [36]. Furthermore, endothelial dysfunction, an early marker of cardiovascular diseases, is more prevalent in preterm infants than in full-term infants [37]. A properly functioning endothelium is crucial for maintaining vascular homeostasis, as it balances factors that regulate both vasoconstriction and vasodilation. Endothelial dysfunction is exacerbated by factors such as dyslipidemia, impaired nutrient metabolism, and atherosclerotic disease. A previous study found that preterm young adults have significantly lower flow-mediated dilation than term-born controls, indicating reduced vascular endothelial function [38]. This suggests an increased risk of cardiovascular disease due to preterm birth. Preterm birth is also linked to an increased risk of developing ischemic heart disease, characterized by reduced blood flow to the heart due to narrowed or blocked coronary arteries [39].

Premature infants have significant challenges in the respiratory system [40]. One contributing factor is the frequent oxygen supply in intensive care management, often due to prematurity or respiratory conditions, which can result in excessive oxygenation of tissues [41]. Mohammadi *et al*. [42] showed that hyperoxia-induced oxidative stress by high oxygen supply can disrupt mitochondrial function, leading to injury in the developing lungs and brain of extremely premature infants. Preterm birth also significantly increases the risk of long-term respiratory diseases. Preterm infants tended to have respiratory distress syndrome due to a deficiency in pulmonary surfactant, crucial for keeping the alveoli in the lungs stable [43]. Previous research evidence showed that the severity of respiratory distress syndrome and mortality rates are negatively correlated to the gestation day, and preterm models showed immature alveolar architecture and minimal surfactant protein-B expression [44]. Bronchopulmonary dysplasia is another common complication in preterm infants. It is characterized by inflammation, scarring, and impaired lung development, ultimately leading to chronic respiratory disease [44, 45]. Prematurity disrupts normal alveolar development, resulting in fewer and larger alveoli and a reduced surface area for gas exchange, which contributes to chronic lung disease [46]. These impairments often necessitate intensive postnatal interventions, including mechanical ventilation, surfactant replacement, and oxygen therapy [45]. Despite advances in perinatal care, such as antenatal corticosteroids and exogenous surfactants, the incidence of chronic lung disease remains higher in preterm infants compared to those born at term [47]. Chronic lung disease, defined by the need for respiratory support or supplemental oxygen at 36 weeks post-conception, is associated with several factors, including the degree of prematurity, severity of initial lung disease, and duration of mechanical ventilation and oxygen therapy [47]. Prematurity interrupts alveolar development, resulting in reduced surface area for gas exchange and leading to chronic lung disease. Preterm infants have immature immune systems, making them more susceptible to respiratory infections, which can exacerbate chronic respiratory issues [48]. Chronic respiratory conditions can lead to reduced physical activity and endurance, with respiratory muscle weakness further impairing muscle growth and function.

Neurological impairments represent another major consequence of preterm birth. Critical phases of brain development occur between 34 and 40 weeks of postmenstrual age [39]. Preterm birth interrupts these processes, increasing the risk of long-term conditions such as cerebral palsy, cognitive impairments, and behavioral disorders [49]. Structural abnormalities in the brain are common and elevate the risk of intraventricular hemorrhage due to fragile germinal matrix vasculature, unstable cerebral blood flow, and coagulation issues [50]. Although most intraventricular hemorrhages are mild, severe cases may result in permanent brain injury. Periventricular leukomalacia, involving white matter damage due to hypoxia or ischemia, is also associated with cerebral palsy and other motor deficits in preterm infants [51]. Long-term challenges may include intellectual disabilities, sensory impairments, growth delays, and social-emotional or learning difficulties [52]. The brain’s high energy demand—accounting for roughly 60% of total metabolism—is particularly taxing in premature infants and contributes to rapid depletion of energy stores, especially during periods of active synaptogenesis and cortical development [53, 54].

Metabolic dysfunction is common in preterm infants. Immature digestive and endocrine systems can lead to impaired insulin secretion and glucose homeostasis, resulting in hypo- or hyperglycemia [55]. Impaired glucose regulation in early life increases the risk of insulin resistance and type 2 diabetes later in life [55]. In addition, altered lipid metabolism can lead to elevated triglyceride and cholesterol levels, further increasing cardiovascular risk [56]. Muscle development may also be compromised due to disrupted nutrient sensing and insulin signaling, resulting in reduced muscle mass and strength [57, 58]. Growth hormone resistance is another issue that can negatively affect physical development [57]. Finally, preterm infants are at increased risk for deficiencies in micronutrients such as iron, calcium, and vitamin D, which can lead to anemia, poor growth, and developmental delays.

### Extrauterine Growth Restriction and Immaturity of Gastrointestinal Tract

Preterm infants are not fully developed in the placenta during gestation and are then born earlier than their designated birth date, resulting in exposure to extra environments with immature digestive and immune systems [59, 60]. In particular, the small intestine has a malfunction in intestinal function and structure, leading to a high risk of inefficiency in nutrient utilization, reduced immune function, and low resistance to disease or viral infection simultaneously [32, 61]. These features are closely related to the vitality and health of preterm infants, making early maturation of intestinal function a critical target in neonatal care strategies [8, 32]. Extrauterine growth restriction (EUGR) commonly occurs in preterm infants after birth with several health challenges [59, 60]. Advancements in neonatal intensive care have significantly improved survival rates, optimizing growth, promoting healthy body composition, and enabling the development of normally functioning organs in premature infants [5, 40]. Without suitable postnatal nutrition, very low birth weight infants struggle with catch-up growth, adversely affecting long-term growth and neurodevelopment [19, 53]. EUGR is a significant issue for very low body weight infants, and their growth retardation results from complex interactions, including health concerns such as endocrine abnormalities, central nervous system damage, difficulties in nutrient intake, and application of medications, or nutritional metabolism [25, 28, 62]. According to Clark *et al*., EUGR significantly affects the postnatal growth of preterm infants, with about 28% for body weight reduction at discharge from the intensive care unit [63]. Chien *et al*., have also shown that neurodevelopmental outcomes, considering cognitive and movement impairments in infants with low birth weights, are closely associated with the severity of their EUGR[64]. In a preterm rat model, EUGR elevated pulmonary arterial pressure in adults and genome-wide epigenetic modifications in pulmonary vascular endothelial cells by increased CpG methylation and H3K27me3 in the Notch1 gene promoter region [65]. Interactions of several environmental factors can exacerbate negative outcomes caused by EUGR. In particular, inadequate early postnatal nutrition is recognized as a leading cause of EUGR. Malnutrition during the critical period of brain development is associated with deficiencies in behavior, learning, and memory, and reduced brain cells [66]. Early and aggressive nutrition aims to minimize cumulative caloric and protein deficits during the acute phase after birth to improve cognitive and neurodevelopmental outcomes related to extra-uterine growth restriction [33, 40].

The stomachs of preterm infants have relatively low acid secretion and decreased proteolytic enzyme activity, resulting in unusually high pH levels and reduced pepsin activity, which can impair protein digestion [67, 68]. This may restrict the absorption of essential amino acids for growth and potentially increase the uptake of allergenic antigens [67]. In addition, insufficient gastric acidity weakens the first line of defense against pathogens, increasing the risk of infection [68, 69]. The intestinal mucosa is also premature, resulting in a structurally weak barrier and significantly lower expression of protective proteins such as mucins, trefoil factor family peptides, and antimicrobial peptides compared to term infants or adults [70, 71]. In particular, the expression of tight junction proteins, critical components for intestinal barrier function, is insufficient, causing a “leaky gut condition characterized by increased intestinal permeability [72]. Increased intestinal permeability allows bacteria and toxins to more easily translocate into the body. Such translocation is a major mechanism underlying systemic inflammatory responses and the development of NEC [72].

Regarding an immunological perspective, preterm infants also have an underdeveloped population of Paneth cells and a lack of the innate immune system, limiting their ability to regulate intestinal microbial balance [73, 74]. The initial colonization of gut microbiota is delayed, and there is often an abnormal predominance of pathogenic Gram-negative bacteria, such as Proteobacteria [11, 15, 16]. This dysbiosis triggers inflammatory responses, leading to epithelial cell damage and disruption of immune homeostasis [9, 15, 16]. Notably, NEC, a severe complication that typically occurs within the first 2–3 weeks of life, is strongly associated with such gut dysbiosis [9, 15, 16]. In addition, the proportions and functions of the four major epithelial cell types—enterocytes, goblet cells, enteroendocrine cells (EECs), and Paneth cells—that actively activate in the intestine remain poorly defined in preterm neonates [8, 32]. Enterocyte efficiency is low, and the number and function of goblet cells, which are responsible for mucus secretion, are insufficient [75]. As a result, the intestinal barrier against pathogens is incomplete. Immune cells, such as lymphocytes, macrophages, and plasma cells, in the lamina propria are not fully established, which limits the function of gut-associated lymphoid tissue [48].

### Intensive Management for Preterm Neonates

Preterm infants need various medical interventions for growth and development, resulting in physiological conditions distinct from those in utero. Non-invasive respiratory support, such as early continuous positive airway pressure (CPAP) and nasal intermittent positive pressure ventilation (NIPPV), is commonly preferred to avoid endotracheal intubation and reduce the risk of bronchopulmonary dysplasia (BPD) [76, 77]. However, when mechanical ventilation becomes necessary, it is associated with increased systemic inflammation and a higher risk of impaired growth and neurodevelopment, highlighting the importance of a timely transition back to non-invasive support [78].

Nutritional management is crucial for early growth and long-term development in preterm infants. Preterm neonates initially require parenteral nutrition until their digestive tract can sufficiently tolerate enteral feeds, as prolonged use of parenteral nutrition can have negative impacts on the development of the liver and digestive tract [79]. Enteral feeding modalities, which deliver milk either intermittently in specific volumes or continuously at a steady rate, have distinct physiological effects and clinical implications [80]. Researchers have compared bolus versus continuous feeding modalities, demonstrating distinct clinical characteristics [79, 80]. In addition to feeding modality, the importance of breast milk feeding is well established because human milk is superior to infant formula in terms of preterm nutrition, primarily due to its bioactive compounds, which include immunomodulatory factors, growth factors, vitamins, and trace elements [81]. These components enhance initial nutritional status and positively influence developmental trajectories in preterm infants. Antibiotic treatment is used to prevent infections in preterm infants. However, antibiotics can also elevate the risk of nephrotoxicity, hepatotoxicity, and antimicrobial resistance, highlighting the importance of careful monitoring and appropriate duration of treatment [82]. Overall, interventions implemented in the neonatal intensive care unit (NICU), including respiratory support, nutritional management strategies, and antibiotic use, significantly impact both immediate survival and the longer-term growth and developmental outcomes of preterm infants.

## Microbial Colonization in Preterm Infants

### Postnatal Progression of Microbial Establishment

The intestinal microbiota in preterm infants develops through a dynamic, sequential process (Table 2) that begins at birth and continues over the first two to three months of life [83, 84]. This early microbial colonization plays a fundamental role in shaping host immunity, metabolic programming, and gut maturation [74]. Shen *et al*.[85] showed that the development of gut microbiota in preterm infants follows a clear temporal pattern that correlates more strongly with postmenstrual age than with postnatal age, delivery mode, or antibiotic exposure, and progresses in defined stages dominated sequentially by *Staphylococcus*, *Enterococcus*, *Enterobacter*, and finally *Bifidobacterium*. In contrast to full-term infants, preterm neonates experience delayed and aberrant microbial succession, with significant clinical implications [86]. Initial colonization typically occurs at the time of birth. In preterm infants, the meconium microbiota is characterized by low diversity and is dominated by Proteobacteria and Firmicutes, with minimal representation of strict anaerobes such as *Bifidobacterium* and *Bacteroides* [74, 87]. Aujoulat *et al*. [88] observed that very preterm infants were largely colonized by facultative anaerobes such as *Staphylococcus*, *Enterococcus*, and Enterobacteriaceae, with diversity developing slowly and remaining distinct from that of term infants. Moreover, differences in birth weight, gestational age, and clinical exposures contribute to variability. Such cohort-specific differences are often difficult to interpret because various factors, including antibiotic exposure, feeding practices, and NICU management, simultaneously affect microbial development. For instance, infants weighing less than 1.1 kg often remain longer in the *Enterococcus*-dominant phase, indicating delayed maturation [89]. By 4 to 6 weeks, several preterm infants can begin to acquire more stable microbiota structures, with increasing *Bifidobacterium* and *Lactobacillus* and a gradual decline in Proteobacteria [86]. Still, extreme prematurity, prolonged antibiotic exposure, or extended NICU stay can sustain dysbiosis and delay convergence toward a term-like microbiota. Although microbial diversity remains low in early life, beta diversity shows convergence across infants over time [84]. Stewart *et al*. [90] further showed that functional maturation proceeds slowly, with early stages dominated by carbohydrate metabolism and later weeks shifting toward amino acid and lipid metabolic functions [86].

After birth, microbial colonization is readily influenced by extrinsic factors, including delivery mode, antibiotic exposure, feeding method, and the neonatal intensive care environment [91, 92]. Zeng *et al*. [93] further emphasized that gut dysbiosis in preterm infants is strongly associated with neonatal intensive care practices, including antibiotic use and parenteral nutrition, particularly in extremely low birth weight infants. Interestingly, the NICU management can influence the shape of early colonization patterns. Infants delivered by cesarean section typically acquire a microbiota enriched in skin‐derived *Staphylococcus*, whereas those delivered vaginally harbor maternal gut–derived taxa such as *Escherichia* and *Bacteroides* [89]. Cesarean delivery has also been linked to delayed establishment of *Bifidobacterium* and reduced *Bacteroides* colonization, whereas vaginal delivery promotes faster microbial succession and higher overall diversity [89]. In addition, continuous exposure to the NICU environment, including medical equipment, surfaces, and staff contact, promotes the establishment of hospital-associated microbes [91]. NICU stay has been associated with persistence of *Klebsiella*, *Enterococcus*, and other *Enterobacteriaceae*, often delaying the transition toward beneficial anaerobes. With these influences, subsequent modulation of the gut microbiota is also strongly influenced by clinical interventions such as antibiotic use and feeding strategies. Intrapartum or postnatal antibiotic administration markedly reduces microbial diversity and delays the expansion of beneficial commensals [89]. Specifically, antibiotics diminish early *Bifidobacterium* colonization and favor expansion of Proteobacteria, particularly *Enterobacteriaceae*, and in some cases increase fungal colonization such as *Candida*. Feeding mode can also affect the microbial colonization: breast milk containing human milk oligosaccharides can support the dominance of *Bifidobacterium*, whereas formula feeding is associated with delayed or altered microbial colonization. Breast milk promotes enrichment of *B. breve* and *B. longum*, which produce short-chain fatty acids that acidify the lumen and suppress pathogens. In contrast, formula-fed infants show higher relative abundance of *Clostridium*, *Enterobacter*, and *Streptococcus*, taxa often linked to necrotizing enterocolitis risk. Moreover, donor milk and formula feeding policies can either accelerate or hinder the functional maturation of the microbiota, influencing the timing of transition toward adult-like communities [92]. Although donor milk confers more protection than formula, pasteurization and processing reduce microbial transfer and alter the metabolic profile compared to breast milk.

Feeding modality has a more distinct influence on microbial development. Tauchi *et al*. [94] also found that breast milk feeding in preterm infants was associated with higher levels of *Bifidobacterium* and lower levels of potentially pathogenic Proteobacteria, reinforcing the protective and microbiota-stabilizing effects of human milk. Direct breastfeeding promotes the transmission of maternal strains such as *B. breve* and *B. longum*, which are not efficiently transferred through formula [95]. Breast milk has also been shown to mitigate antibiotic-induced disruptions in microbial development [74]. In addition, fecal samples from preterm infants reveal strong taxonomic overlap across time, suggesting that early microbial imprinting can persist for several weeks [16, 84, 96]. Studies that follow babies over time show that the amount of weight a baby gains and the duration since pregnancy began are better indicators of gut bacteria development than simply counting the days after birth [83, 89]. Despite individual variation, the overall trajectory in preterm infants reveals delayed establishment of strict anaerobes, high initial dominance by facultative pathogens, and prolonged dysbiosis in the absence of supportive interventions [97, 98]. These findings highlight the neonatal period as a critical window in which targeted interventions could redirect microbial development and improve long-term outcomes.

### Microbial Composition and Diversity in Preterm Infants

The intestinal microbiota of preterm infants differs significantly from that of term infants, influenced by developmental immaturity and postnatal management. These differences lead to taxonomic shifts, altered functional capabilities, and modified metabolic activities, carrying profound implications for the development of the immune system and long-term health outcomes. Preterm infants predominantly exhibit intestinal microbiota characterized by facultative anaerobes, notably members of Proteobacteria such as Enterobacteriaceae, *Klebsiella*, and *Escherichia coli*, accompanied by reduced colonization by obligate anaerobes including *Bifidobacterium*, *Bacteroides*, and *Clostridia* [87, 96, 99]. Table 3 summarizes key features of gut microbiota development based on previous findings. Preterm piglets are a valuable model for prematurity study, as they have also been shown reduced microbial diversity, delayed anaerobe colonization, and epigenetic alterations in immune and metabolic genes influenced by feeding strategies [100, 101]. The dysbiosis results from delayed microbial succession, which is exacerbated by clinical practices such as cesarean delivery, antibiotic use, and limited breastfeeding opportunities inherent in neonatal intensive care units [97, 102].

Longitudinal studies consistently indicate lower microbial diversity in preterm infants, particularly during the initial postnatal weeks. Chu *et al*., [103] reported a significant reduction in Shannon diversity indices by 0.45 units in preterm infants compared to term infants within the first 10 days post-birth, a phenomenon independent of feeding modality or antibiotic exposure but closely associated with the length of NICU management. Although alpha-diversity tends to increase over time, it often remains lower than that observed in term infants even beyond early childhood [96, 104, 105]. Beta-diversity, which indicates differences in microbial composition between individuals, initially exhibits considerable variability among preterm infants due to diverse clinical interventions and environmental exposures, but typically converges as infants mature [84, 96]. Enterotype transitions, characterized by shifts from dominance of *Staphylococcus* or *Enterobacter* to stable *Bifidobacterium*- or *Bacteroides*-rich profiles, occur later in preterm infants, particularly in cases of extreme prematurity or prolonged antibiotic exposure [83, 86, 91]. Importantly, although microbial diversity and composition tend to stabilize over time, distinct microbial imprints persist into later childhood, characterized by delayed acquisition of adult-like microbiota and associated functional maturity [104, 105]. In addition, maternal microbiota during pregnancy has been shown to influence neonatal microbiota development, with mothers delivering preterm showing reduced gut diversity and decreased levels of beneficial bacteria such as *Bifidobacterium* and *Streptococcus* compared to mothers delivering at term [99]. Therefore, these findings underscore the complex interplay of developmental immaturity, clinical practices, and environmental exposures that shape the microbiota composition and diversity in preterm infants. Persistent dysbiosis and altered maturation pathways highlight the critical importance of targeted interventions during neonatal care to promote optimal microbial development and support long-term physiological health.

Feeding practices and probiotic supplementation have a significant impact on the microbiota. Breastfeeding promotes beneficial colonization by *B. breve* and *B. longum*, enhancing carbohydrate metabolism pathways [95, 106]. In contrast, formula feeding and cow’s milk-based fortifiers may encourage Proteobacteria proliferation and reduce microbial evenness [106]. Although probiotic interventions have demonstrated benefits such as increased SCFAs, lowered intestinal pH, and reduced abundance of pathogenic taxa, previous studies highlight variability in outcomes influenced by strain, dosage, and administration timing [16, 107]. According to previous studies, supplementation of *Lactobacillus rhamnosus* has been associated with reduced NEC incidence and improved barrier function [108, 109], whereas *B. breve* supplementation showed more modest benefits, with inconsistent effects on NEC prevention but improvements in metabolic outcomes for preterm infants [110, 111]. Probiotic effects are widely considered strain-specific, and recent meta-analyses confirm that *L. rhamnosus* GG used as a single strain reduced NEC risk, but showed no significant effects on late-onset sepsis or mortality, whereas observational studies did not replicate these benefits [112]. Despite these promising findings, results from randomized controlled trials remain inconsistent. Some large trials and network meta-analyses report significant reductions in NEC and mortality with certain probiotic regimens, whereas others show no clear benefit, reflecting heterogeneity in trial design, product formulations, and patient populations [113]. A review of probiotic-related sepsis in preterm infants also demonstrated that although probiotics may reduce the risk of NEC and death, but rare cases of probiotic-associated sepsis and fatal infections from contaminated products highlight safety concerns, highlighting the need for strict quality control and careful monitoring in extremely preterm infants [114].

## Roles and Health of Intestinal Microbiota in Preterm Infants

### Immune and Intestinal Development

In preterm infants, an underdeveloped gastrointestinal tract is often accompanied by gut dysbiosis, disrupting the synchrony between immune maturation and intestinal epithelial development [102, 115]. Preterm infants often show microbiome compositions characterized by reduced microbial diversity and dominance of facultative anaerobes, such as *Klebsiella*, *Escherichia*, *Staphylococcus*, and *Enterococcus*, significantly impairing mucosal defense and barrier integrity [115]. These bacteria are also known to produce little or no short-chain fatty acids and are primarily associated with pathogenicity, dysbiosis, and inflammation. Microbial metabolites, such as short-chain fatty acids (SCFAs), play a pivotal role in regulating immune responses and reinforcing epithelial defense [116]. Butyrate has been shown to strengthen tight junction integrity by upregulating claudin-1, occludin, and ZO-1 gene expression and promoting oxygen-sensing pathways through GPR43-mediated HIF-1α stabilization [117, 118]. Butyrate suppresses histone deacetylases (HDACs), enhancing FOXP3 transcription and thereby promoting the expansion of regulatory T cells [119]. It could also be related to IL-10 production, suppressing IL-6 and TNF-α, which are two cytokines frequently elevated in preterm infants with intestinal inflammation [120]. Intestinal cell types, such as Paneth cells and goblet cells, exhibit both reduced number and function in preterm infants [121]. Paneth cells, located at the base of intestinal crypts, are crucial for secreting antimicrobial peptides such as lysozyme, defensins, and RegIIIγ, which help to control microbial colonization and maintain intestinal homeostasis [122]. In preterm infants, Paneth cells display delayed differentiation, decreased granule formation, and significantly reduced antimicrobial peptide secretion [123]. Goblet cells, responsible for mucus production, also exhibit impaired functionality in preterm neonates [124]. The mucus layer produced by goblet cells serves as a primary physical barrier against microbial invasion [125]. Reduced mucus secretion, decreased mucin gene (MUC2) expression, and altered mucus composition in preterm infants lead to compromised barrier function and heightened susceptibility to pathogens and inflammatory stimuli [124]. This combined impairment of Paneth and goblet cells intensifies vulnerability to microbial invasion and inflammation, contributing to a higher incidence of severe intestinal conditions, such as NEC, in preterm infants [115].

Gut microbiota in full-term infants is more effective at inducing tolerogenic dendritic cells and anti-inflammatory immune profiles different from preterm neonates [126]. Dysbiosis in preterm infants often features Proteobacteria dominance and reduced microbial diversity, both of which correlate with heightened Th17/Th1 immune responses and impaired cell development [11, 126]. Elevated levels of IL-6 and IL-8 in serum samples of infants with NEC further highlight the pro-inflammatory bias associated with a disrupted microbiota [126]. Conversely, colonization by *B. breve* has been linked to enhanced mucin gene expression (MUC2, TFF3), improved barrier function, and a more regulated immune response [127]. Probiotic supplementation with beneficial strains has shown potential to enhance mucosal immunity, stimulate regulatory T-cell differentiation, increase IgA production, and restore barrier function by improving epithelial proliferation and differentiation [128, 129]. Moreover, breastmilk provides critical bioactive components such as milk oligosaccharides, immunoglobulins, and lactoferrin, which facilitate beneficial microbial colonization and reinforce intestinal barrier integrity [81, 130]. However, preterm infants frequently have limited capacity for breastfeeding, thus significantly reducing their intake of the biological benefits from maternal nutrition. This restricted access to breastfeeding exacerbates gut dysbiosis and further impairs immune and intestinal development. These findings collectively indicate that microbial signals are integral to coordinating both physical and immunological maturation of the neonatal gut, and interventions that support beneficial colonization may offer long-term protective benefits in premature populations.

Necrotizing enterocolitis and neonatal sepsis are two of the most devastating complications associated with prematurity, both closely linked to intestinal dysbiosis. NEC is predominantly observed in very-low-birth-weight infants, with a mortality rate reaching up to 30% in severe cases [131]. A hallmark of NEC pathogenesis is the aberrant activation of innate immune signaling in the immature intestine, especially through Toll-like receptor 4 (TLR4) in response to lipopolysaccharide (LPS) derived from Gram-negative bacteria. This leads to exaggerated inflammatory cascades, epithelial injury, and compromised barrier function, ultimately resulting in intestinal necrosis [131-133]. Previous studies have demonstrated that TLR4 is overexpressed in the premature gut and its activation by endotoxin impairs intestinal perfusion via endothelial nitric oxide synthase signaling [134]. Additionally, mice deficient in TLR4 show resistance to NEC, underscoring its central role in disease onset [132]. Clinically, NEC is preceded by a characteristic shift in the gut microbiota that typically a dominance of Proteobacteria and a depletion of Firmicutes and *Bifidobacterium* occurring 2–3 weeks before symptom onset [135, 136]. This pattern was also observed in a previous study, which showed that neonates who developed NEC or feeding intolerance had reduced microbial diversity and significantly lower levels of *Bifidobacterium* and *Streptococcus* in their fecal samples [84]. These microbial patterns are further exacerbated by clinical practices such as prolonged antibiotic use, cesarean section, and formula feeding, which suppress commensal colonization and amplify pathogenic proliferation [131]. Neonatal sepsis shares overlapping microbial and immunological features with NEC. Intestinal dysbiosis facilitates the translocation of gut-derived pathogens into the bloodstream, especially under conditions of barrier disruption. In a multi-site prospective study, over 80% of bloodstream isolates in septic neonates matched strains found in their stool samples, indicating gut-origin bacteremia [137]. Antibiotic-induced depletion of protective commensals such as Bifidobacterium and Lactobacillus allows for opportunistic expansion of Enterobacteriaceae, increasing sepsis risk [131]. Furthermore, the presence of specific microbial signatures, including elevated abundance of *Klebsiella* and *Enterococcus* prior to sepsis onset, has been reported in stool samples of infants who subsequently developed sepsis [138]. This suggests the potential of gut microbial profiling as a predictive biomarker for both NEC and sepsis.

### Gut-Brain Axis and Neurodevelopment

**Fig. 1:**
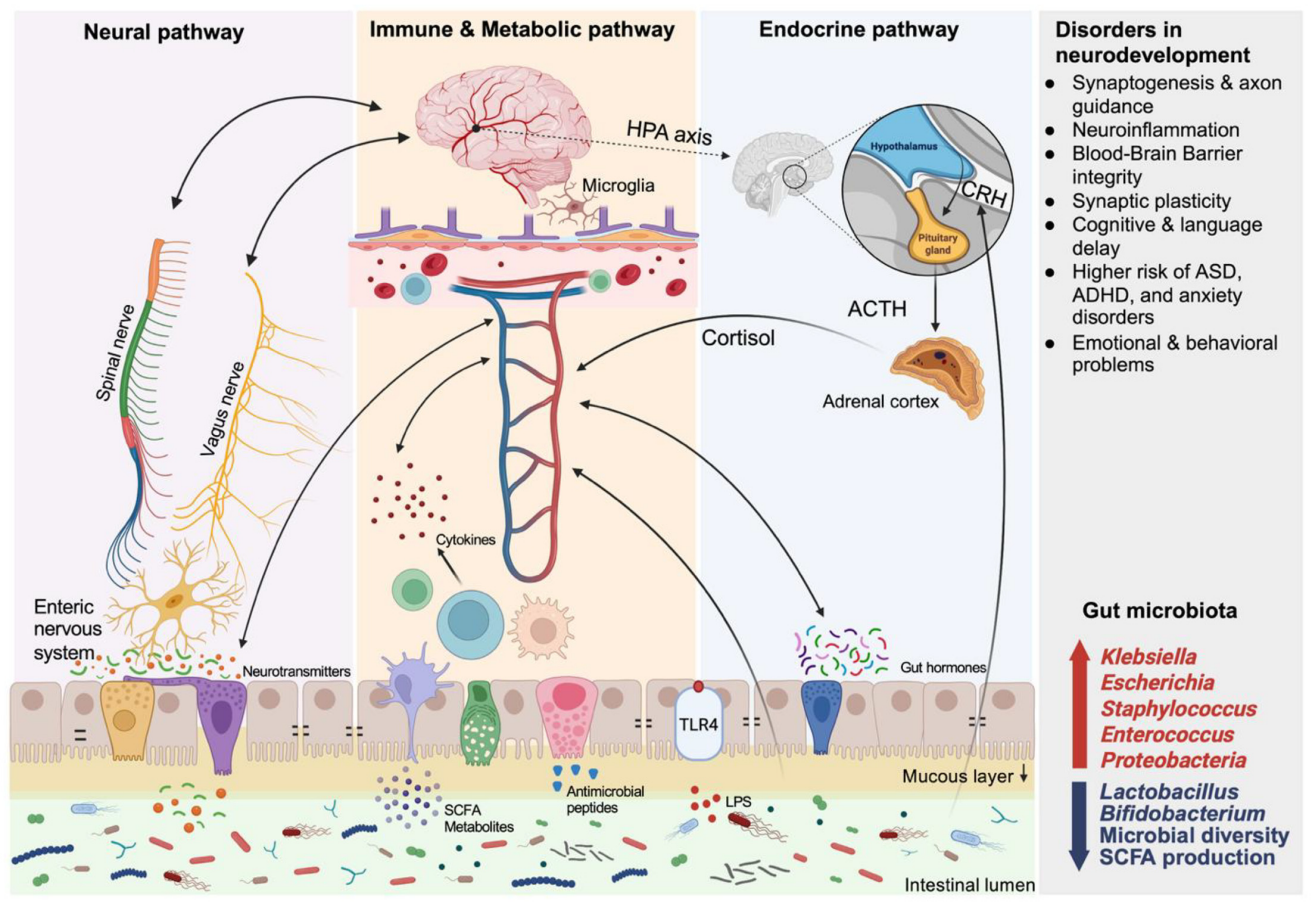
Gut–brain axis mechanisms in preterm infants. The gut–brain axis in preterm infants involves the neural pathway via the vagus and spinal nerves, the immune–metabolic pathway related to cytokines, microglial activation, blood– brain barrier (BBB) regulation, and short-chain fatty acids (SCFAs), and the endocrine pathway mediated by the hypothalamic–pituitary–adrenal (HPA) axis and cortisol release. Dysbiosis, marked by an increased abundance of several bacteria (*Klebsiella*, *Escherichia*, *Staphylococcus*, *Enterococcus*, and Proteobacteria) and a reduction in beneficial taxa (*Lactobacillus* and *Bifidobacterium*), microbial diversity, and SCFA production, further compromises intestinal and neurodevelopment.

The development of the gut-brain axis during early life plays a critical role in shaping long-term neuro-developmental outcomes, especially in preterm infants (Fig. 1). Communication between the gut and the brain occurs through three interconnected pathways: the neural pathway mediated by the vagus and spinal nerves, the immune and metabolic pathway involved in cytokines, microglial activation, blood–brain barrier regulation, and microbial metabolites such as SCFAs, and the endocrine pathway by the hypothalamic–pituitary–adrenal (HPA) axis and cortisol release. Immaturity or dysregulation of these pathways in preterm infants disrupts critical neurodevelopmental processes, resulting in impaired synaptogenesis, heightened neuroinflammation, reduced blood–brain barrier integrity, and diminished synaptic plasticity. Such alterations increase susceptibility to cognitive and language delays, autism spectrum disorder (ASD), attention-deficit/hyperactivity disorder (ADHD), anxiety, and a range of emotional or behavioral difficulties. These infants often exhibit delayed colonization of commensals, reduced microbial diversity, and increased proportions of pathobionts. Gut microbiota can be a key factor in this process, as preterm infants typically display an overrepresentation of pathobionts such as *Klebsiella*, *Escherichia*, *Staphylococcus*, *Enterococcus*, and members of Proteobacteria, alongside a reduction in beneficial taxa such as *Lactobacillus* and *Bifidobacterium*. This gut dysbiosis, characterized by decreased microbial diversity and reduced SCFA production, further compromises both intestinal and neurodevelopmental health. Such dysbiosis has been linked to neurodevelopmental impairments, including autism spectrum disorder, ADHD, and anxiety [139]. Lu *et al*. [140] demonstrated that transplanting fecal microbiota from preterm infants of lower postmenstrual age into germ-free mice led to significantly reduced associative fear learning and altered gene expression in the brain. Similarly, Carlson *et al*. [141] found that higher microbial diversity in infants under one year of age was positively correlated with enhanced cognitive scores based on the Bayley Scales of Infant and Toddler Development, a standardized assessment that evaluates cognitive, language, and motor skills in infants and toddlers.

The gut microbiota modulates brain development via immune regulation, microbial metabolite signaling, and neuroinflammatory pathways [142]. Among these, short-chain fatty acids have been shown to influence blood-brain barrier integrity, microglial maturation, and synaptic plasticity [143, 144]. Lu *et al*. [144] reported that mice receiving preterm microbiota displayed reduced brain IGF-1 levels, lower NeuN expression, and diminished myelin basic protein (MBP), indicating impaired brain maturation. In addition, Wang *et al*. [145] used Mendelian randomization analysis to reveal that SCFA-producing Faecalibacterium is causally associated with a reduced risk of ADHD, further supporting a role for microbial metabolites in neurodevelopment. Early-life dysbiosis also impacts tryptophan metabolism and serotonin biosynthesis, both of which influence synaptogenesis and axon guidance [139]. Mhanna *et al*. [146] reviewed how gut microbes regulate biosynthetic pathways of neurotransmitters such as GABA, dopamine, and serotonin, implicating these in the pathophysiology of ASD and anxiety. Beghetti *et al*. [139] specifically noted that dysbiosis in preterm infants suppresses tryptophan–serotonin pathways, thereby disrupting neural connectivity and neuron maturation.

The physiological architecture of the gut–brain axis includes peripheral and central components such as the enteric nervous system, vagus nerve, HPA axis, and limbic structures. In preterm neonates, stress-related signals originating in the gut may be transduced via vagal or humoral routes, activating the HPA axis and influencing emotional regulation circuits. Environmental factors such as maternal separation, intensive care unit stress, or intestinal inflammation can exacerbate these responses, particularly affecting the hippocampus and long-range neural connectivity.

The connection between gut dysbiosis and neuroinflammation is especially evident in models of NEC. Xia and Claud [142] demonstrated that gut-derived IFN-γ-producing CD4^+^ T cells migrate to the brain, where they activate microglia, leading to neurotoxicity and white matter injury. Previous studies [131, 147] further described that dysbiosis in preterm neonates impairs myelination and triggers microglial activation, contributing to impaired motor outcomes. Sherman *et al*. [148] also reported that inflammatory cytokines and microbial toxicants derived from the dysbiosis can enter systemic circulation and reach the brain, leading to white matter injury and long-term neurodevelopmental damage even in the absence of intracranial hemorrhage. These microbe–immune–neural interactions also influence stress circuitry through the hypothalamic-pituitary-adrenal (HPA) axis. Beghetti *et al*. [139] suggested that preterm infants experiencing early dysbiosis exhibit HPA hyperactivation, potentially leading to long-term emotional and behavioral dysregulation. Dysregulation of gut hormones and neuropeptides (*e.g.*, ghrelin, peptide YY, GLP-1) in response to gut dysbiosis can exacerbate these disorders [148]. Krupa-Kotara *et al*. [149] provided evidence that such dysbiosis-induced alterations in stress circuits can be reversed through probiotic intervention.

Human studies have shown specific microbial patterns, such as increased *Bacteroides* and *Lachnospiraceae* abundance, to improve cognitive and language scores at two years of age [150]. Prtty *et al*. [150] reported that very preterm infants with higher alpha diversity and lower Proteobacteria levels in early fecal samples showed significantly higher Bayley cognitive and language scores at age two. Colonization by beneficial commensals such as *Faecalibacterium prausnitzii* and *Bacteroides thetaiotaomicron* promotes mucosal barrier integrity and anti-inflammatory signaling, thereby protecting the developing brain from microbial translocation and neuroimmune stress [148]. Moreover, maternal and neonatal probiotic supplementation has demonstrated neurodevelopmental benefits in animal models, suggesting possible benefits in preterm human infants [139]. Probiotic bacteria can inhibit proinflammatory signaling cascades (*e.g.*, TNF-α and NF-κB), enhance IL-10 production, and reduce gut-derived systemic inflammation, ultimately preventing brain injury in preterm models.

## Conclusion

**Fig. 2:**
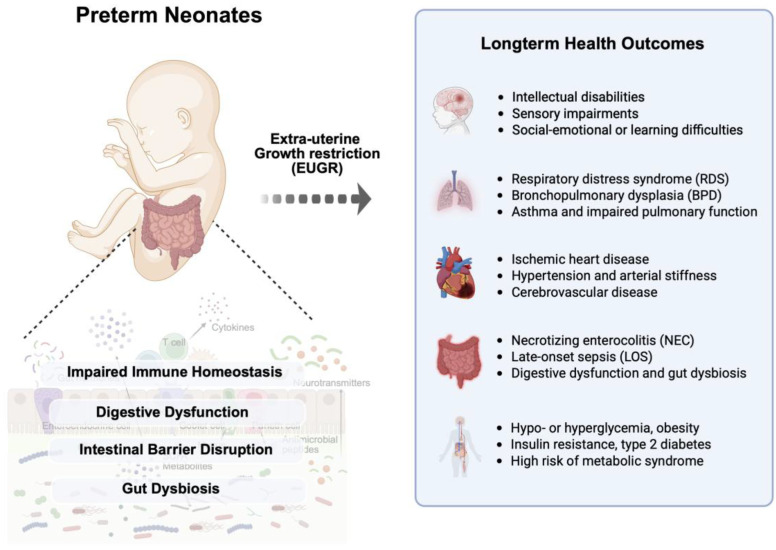
Impact of intestinal immaturity in preterm infants on growth restriction and long-term health disorders. Impaired maturation of the digestive system in preterm infants leads to gut dysbiosis and increases susceptibility to extra-uterine growth restriction (EUGR). These alterations contribute to elevated risks of neurodevelopmental, respiratory, cardiovascular, gastrointestinal, and metabolic disorders.

In conclusion, gut dysbiosis is not only an acute challenge of prematurity but also an underlying cause of long-term health outcomes (Fig. 2). These microbial alterations increase the risk of severe neonatal diseases, including NEC and sepsis, and contribute to persistent neurodevelopmental and metabolic disorders. Commensal microbes and their metabolites play a pivotal role in epithelial maturation, immune regulation, and gut–brain signaling. Therefore, advancing neonatal care will require precision strategies that integrate microbial profiling with targeted nutritional interventions and a deeper understanding of host–microbe interactions to promote healthier outcomes in preterm infants.

## Figures and Tables

**Table 1:** Clinical and developmental challenges in preterm neonates.

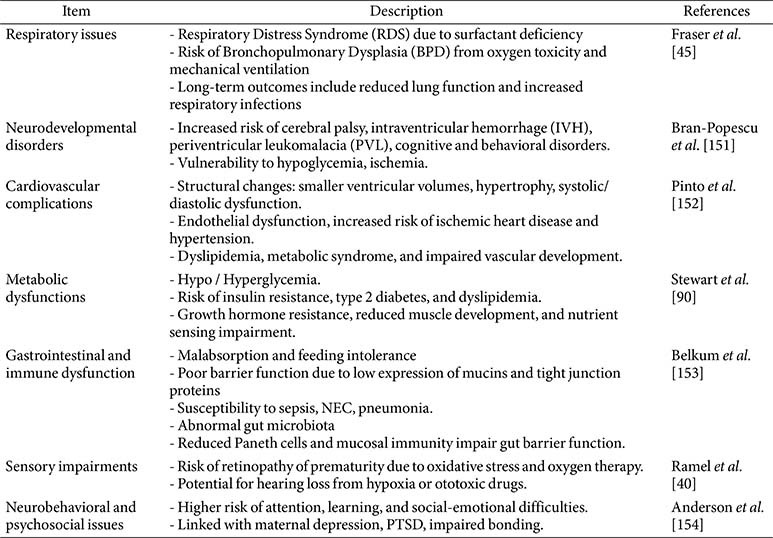

**Table 2:** Postnatal progression of fecal microbiota composition in preterm infants.

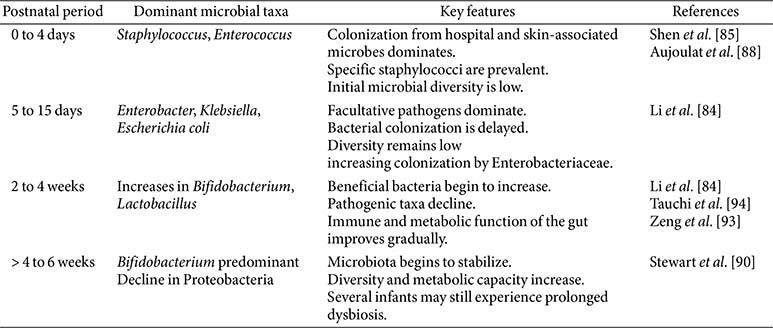

**Table 3:** Preterm gut microbiota development in humans and piglet models.

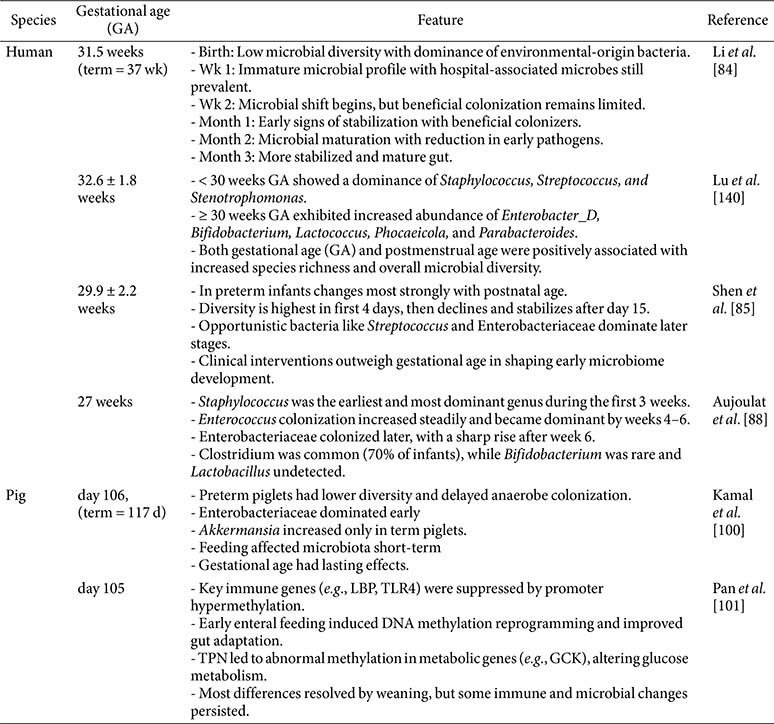

## References

[1] Ohuma EO, Moller AB, Bradley E, Chakwera S, Hussain-Alkhateeb L, Lewin A (2023). National, regional, and global estimates of preterm birth in 2020, with trends from 2010: a systematic analysis. Lancet.

[2] Inskeep EK (2004). Preovulatory, postovulatory, and postmaternal recognition effects of concentrations of progesterone on embryonic survival in the cow1,2. J. Anim. Sci..

[3] Swanson AM, David AL (2015). Animal models of fetal growth restriction: considerations for translational medicine. Placenta.

[4] Lammertink F, Vinkers CH, Tataranno ML, Benders M (2020). Premature birth and developmental programming: mechanisms of resilience and vulnerability. Front. Psychiatry.

[5] Ramel SE, Gray HL, Ode KL, Younge N, Georgieff MK, Demerath EW (2011). Body composition changes in preterm infants following hospital discharge: comparison with term infants. J. Pediatr. Gastroenterol. Nutr..

[6] Daskalakis G, Psarris A, Koutras A, Fasoulakis Z, Prokopakis I, Varthaliti A (2023). Maternal infection and preterm birth: from molecular basis to clinical implications. Children (Basel).

[7] Takiishi T, Fenero CIM, Câmara NOS (2017). Intestinal barrier and gut microbiota: shaping our immune responses throughout life. Tissue Barriers.

[8] Indrio F, Neu J, Pettoello-Mantovani M, Marchese F, Martini S, Salatto A (2022). Development of the gastrointestinal tract in newborns as a challenge for an appropriate nutrition: a narrative review. Nutrients.

[9] Duess JW, Sampah ME, Lopez CM, Tsuboi K, Scheese DJ, Sodhi CP (2023). Necrotizing enterocolitis, gut microbes, and sepsis. Gut Microbes.

[10] Huang H, Jiang J, Wang X, Jiang K, Cao H (2024). Exposure to prescribed medication in early life and impacts on gut microbiota and disease development. EClinicalMedicine.

[11] Groer MW, Luciano AA, Dishaw LJ, Ashmeade TL, Miller E, Gilbert JA (2014). Development of the preterm infant gut microbiome: a research priority. Microbiome.

[12] Lee D, Goh TW, Kang MG, Choi HJ, Yeo SY, Yang J (2022). Perspectives and advances in probiotics and the gut microbiome in companion animals. J. Anim. Sci. Technol..

[13] Mun D, Kim H, Shin M, Ryu S, Song M, Oh S (2021). Decoding the intestinal microbiota repertoire of sow and weaned pigs using culturomic and metagenomic approaches. J. Anim. Sci. Technol..

[14] Kang A, Eor JY, Lee J, Kwak MJ, Lee DJ, Seo E (2025). *Lacticaseibacillus casei* IDCC 3451 alleviates cognitive and behavioral functions by reshaping the gut microbiome and regulating intestinal barrier integrity in chronic stress animal models. Curr. Res. Food Sci..

[15] Ma Y, Peng X, Zhang J, Zhu Y, Huang R, Li G (2024). Gut microbiota in preterm infants with late-onset sepsis and pneumonia: a pilot case-control study. BMC Microbiol..

[16] Yang S, He J, Shi J, Xie L, Liu Y, Xiong Y (2024). Characteristics of intestinal microbiota in preterm infants and the effects of probiotic supplementation on the microbiota. Front. Microbiol..

[17] Morrison KM, Ramsingh L, Gunn E, Streiner D, Van Lieshout R, Boyle M (2016). Cardiometabolic health in adults born premature with extremely low birth weight. Pediatrics.

[18] Roggero P, Giann ML, Amato O, Orsi A, Piemontese P, Morlacchi L (2009). Is term newborn body composition being achieved postnatally in preterm infants?. Early Hum. Dev..

[19] Johnson MJ, Wootton SA, Leaf AA, Jackson AA (2012). Preterm birth and body composition at term equivalent age: a systematic review and meta-analysis. Pediatrics.

[20] Olhager E, Törnqvist C (2014). Body composition in late preterm infants in the first 10 days of life and at full term. Acta Paediatr..

[21] Viswanathan S, Thoene M, Alja'nini Z, Alur P, McNelis K (2025). Body composition in preterm infants: current insights and emerging perspectives. Children (Basel).

[22] Möllers LS, Yousuf EI, Hamatschek C, Morrison KM, Hermanussen M, Fusch C (2022). Metabolic-endocrine disruption due to preterm birth impacts growth, body composition, and neonatal outcome. Pediatr. Res..

[23] Chen Y, Michalak M, Agellon LB (2018). Importance of nutrients and nutrient metabolism on human health. Yale J. Biol. Med..

[24] Kamity R, Kapavarapu PK, Chandel A (2021). Feeding problems and long-term outcomes in preterm infants-a systematic approach to evaluation and management. Children (Basel).

[25] Cho J, Chien LC, Holditch-Davis D (2021). Associations between hormonal biomarkers and preterm infant health and development during the first 2 years after birth. Biol. Res. Nurs..

[26] Choi Y, Kang A, Kang MG, Lee DJ, Kyoung D, Kwak MJ, *et al*. 2025. Exploring synergistic effect of bacteriophages with probiotics against multidrug resistant *Salmonella* Typhimurium in a simulated chicken gastrointestinal system using metagenomic- and culturomic approaches. *J. Anim. Sci. Technol.* https://doi.org/10.5187/jast.2025.e30. 10.5187/jast.2025.e30

[27] Ng PC, Lam CW, Lee CH, Wong GW, Fok TF, Chan IH (2000). Leptin and metabolic hormones in preterm newborns. Arch. Dis. Child Fetal Neonatal. Ed..

[28] Casirati A, Somaschini A, Perrone M, Vandoni G, Sebastiani F, Montagna E (2022). Preterm birth and metabolic implications on later life: a narrative review focused on body composition. Front. Nutr..

[29] Suryawan A, Naberhuis J, Rudar M, Fiorotto M, Davis T (2021). Regulation of akt signaling in skeletal muscle is altered by prematurity in a neonatal piglet model. Curr. Dev. Nutr..

[30] Naberhuis JK, Suryawan A, Nguyen HV, Hernandez-Garcia A, Cruz SM, Lau PE (2019). Prematurity blunts the feedinginduced stimulation of translation initiation signaling and protein synthesis in muscle of neonatal piglets. Am. J. Physiol. Endocrinol. Metab..

[31] Hay WW, Brown LD, Denne SC (2014). Energy requirements, protein-energy metabolism and balance, and carbohydrates in preterm infants. World Rev. Nutr. Diet..

[32] Jacobi SK, Odle J (2012). Nutritional factors influencing intestinal health of the neonate. Adv. Nutr..

[33] Ibrahim HM, Jeroudi MA, Baier RJ, Dhanireddy R, Krouskop RW (2004). Aggressive early total parental nutrition in low-birthweight infants. J. Perinatol..

[34] Kumar VHS (2022). Cardiovascular morbidities in adults born preterm: getting to the heart of the matter!. Children (Basel).

[35] Raju TNK, Buist AS, Blaisdell CJ, Moxey-Mims M, Saigal S (2017). Adults born preterm: a review of general health and systemspecific outcomes. Acta Paediatr..

[36] Sixtus RP, Dyson RM, Gray CL. 2024. Impact of prematurity on lifelong cardiovascular health: structural and functional considerations. *NPJ Cardiovascular Health* 1. https://doi.org/10.1038/s44325-024-00002-0. 10.1038/s44325-024-00002-0 41036413 PMC12479354

[37] Amelio GS, Provitera L, Raffaeli G, Tripodi M, Amodeo I, Gulden S (2022). Endothelial dysfunction in preterm infants: the hidden legacy of uteroplacental pathologies. Front. Pediatr..

[38] Engan B, Engan M, Greve G, Vollsæter M, Hufthammer KO, Leirgul E (2021). Vascular endothelial function assessed by flowmediated vasodilatation in young adults born very preterm or with extremely low birthweight: a regional cohort study. Front. Pediatr..

[39] Lewandowski AJ, Levy PT, Bates ML, McNamara PJ, Nuyt AM, Goss KN (2020). Impact of the vulnerable preterm heart and circulation on adult cardiovascular disease risk. Hypertension.

[40] Ramel SE, Brown LD, Georgieff MK (2014). The impact of neonatal illness on nutritional requirements-one size does not fit all. Curr. Pediatr. Rep..

[41] Fairchild K, Mohr M, Paget-Brown A, Tabacaru C, Lake D, Delos J (2016). Clinical associations of immature breathing in preterm infants: part 1-central apnea. Pediatr. Res..

[42] Mohammadi A, Higazy R, Gauda EB (2022). PGC-1α activity and mitochondrial dysfunction in preterm infants. Front. Physiol..

[43] Fraser J, Walls M, McGuire W (2004). Respiratory complications of preterm birth. BMJ.

[44] Caminita F, van der Merwe M, Hance B, Krishnan R, Miller S, Buddington K (2015). A preterm pig model of lung immaturity and spontaneous infant respiratory distress syndrome. Am. J. Physiol. Lung Cell. Mol. Physiol..

[45] Fraser J, Walls M, McGuire W (2005). ABC of preterm birth: respiratory complications of preterm birth. BMJ.

[46] Bourbon J, Boucherat O, Chailley-Heu B, Delacourt C (2005). Control mechanisms of lung alveolar development and their disorders in bronchopulmonary dysplasia. Pediatr. Res..

[47] Christ LA, Sucre JM, Frank DB (2019). Lung disease and pulmonary hypertension in the premature infant. Prog. Pediatr. Cardiol..

[48] Melville JM, Moss TJ (2013). The immune consequences of preterm birth. Front. Neurosci..

[49] Yates N, Gunn AJ, Bennet L, Dhillon SK, Davidson JO (2021). Preventing brain injury in the preterm infant-current controversies and potential therapies. Int. J. Mol. Sci..

[50] Ballabh P (2010). Intraventricular hemorrhage in premature infants: mechanism of disease. Pediatr. Res..

[51] Volpe JJ (2001). Neurobiology of periventricular leukomalacia in the premature infant. Pediatr. Res..

[52] Shaw RJ, Givrad S, Poe C, Loi EC, Hoge MK, Scala M (2023). Neurodevelopmental, mental health, and parenting issues in preterm infants. Children (Basel).

[53] Brummelte S, Grunau RE, Chau V, Poskitt KJ, Brant R, Vinall J (2012). Procedural pain and brain development in premature newborns. Ann. Neurol..

[54] Kuzawa CW (1998). Adipose tissue in human infancy and childhood: an evolutionary perspective. Am. J. Phys. Anthropol. Suppl.

[55] Sharma A, Davis A, Shekhawat PS (2017). Hypoglycemia in the preterm neonate: etiopathogenesis, diagnosis, management and long-term outcomes. Transl. Pediatr..

[56] Crump C, Sundquist J, Sundquist K (2019). Association of preterm birth with lipid disorders in early adulthood: a swedish cohort study. PLoS Med..

[57] Dobson NL, Levitt DE, Luk HY, Vellers HL (2024). Adverse skeletal muscle adaptations in individuals born preterm-a comprehensive review. Curr. Issues Mol. Biol..

[58] Park J, Moon SS, Song S, Cheng H, Im C, Du L (2024). Comparative review of muscle fiber characteristics between porcine skeletal muscles. J. Anim. Sci. Technol..

[59] Tozzi MG, Moscuzza F, Michelucci A, Lorenzoni F, Cosini C, Ciantelli M (2018). ExtraUterine Growth Restriction (EUGR) in preterm infants: growth patterns, nutrition, and epigenetic markers. A pilot study. Front. Pediatr..

[60] Peila C, Spada E, Giuliani F, Maiocco G, Raia M, Cresi F (2020). Extrauterine growth restriction: definitions and predictability of outcomes in a cohort of very low birth weight infants or preterm neonates. Nutrients.

[61] Davis EC, Wang M, Donovan SM (2017). The role of early life nutrition in the establishment of gastrointestinal microbial composition and function. Gut Microbes.

[62] Rodríguez-Cano AM, Calzada-Mendoza CC, Estrada-Gutierrez G, Mendoza-Ortega JA, Perichart-Perera O (2020). Nutrients, mitochondrial function, and perinatal health. Nutrients.

[63] Clark RH, Thomas P, Peabody J (2003). Extrauterine growth restriction remains a serious problem in prematurely born neonates. Pediatrics.

[64] Chien HC, Chen CH, Wang TM, Hsu YC, Lin MC (2018). Neurodevelopmental outcomes of infants with very low birth weights are associated with the severity of their extra-uterine growth retardation. Pediatr. Neonatol..

[65] Tang LL, Zhang LY, Lao LJ, Hu QY, Gu WZ, Fu LC (2015). Epigenetics of Notch1 regulation in pulmonary microvascular rarefaction following extrauterine growth restriction. Respir. Res..

[66] Grantham-McGregor S (1995). A review of studies of the effect of severe malnutrition on mental development. J. Nutr..

[67] Demers-Mathieu V, Qu Y, Underwood MA, Borghese R, Dallas DC (2018). Premature infants have lower gastric digestion capacity for human milk proteins than term infants. J. Pediatr Gastroenterol. Nutr..

[68] Neu J (1996). Necrotizing enterocolitis: the search for a unifying pathogenic theory leading to prevention. Pediatr. Clin. North Am..

[69] Manzoni P, García Sánchez R, Meyer M, Stolfi I, Pugni L, Messner H (2018). Exposure to gastric acid inhibitors increases the risk of infection in preterm very low birth weight infants but concomitant administration of lactoferrin counteracts this effect. J. Pediatr..

[70] Demers-Mathieu V (2022). The immature intestinal epithelial cells in preterm infants play a role in the necrotizing enterocolitis pathogenesis: a review. Health Sci. Rev..

[71] Golubkova A, Hunter CJ (2023). Development of the neonatal intestinal barrier, microbiome, and susceptibility to NEC. Microorganisms.

[72] Khoshbin K, Camilleri M (2020). Effects of dietary components on intestinal permeability in health and disease. Am. J. Physiol. Gastrointest Liver Physiol..

[73] Brown JA, Bashir H, Zeng MY (2025). Lifelong partners: gut microbiota-immune cell interactions from infancy to old age. Mucosal Immunol..

[74] Xiang Q, Yan X, Shi W, Li H, Zhou K (2023). Early gut microbiota intervention in premature infants: application perspectives. J. Adv. Res..

[75] Buisine MP, Devisme L, Savidge TC, Gespach C, Gosselin B, Porchet N (1998). Mucin gene expression in human embryonic and fetal intestine. Gut.

[76] Lemyre B, Deguise MO, Benson P, Kirpalani H, Ekhaguere OA, Davis PG (2023). Early nasal intermittent positive pressure ventilation (NIPPV) versus early nasal continuous positive airway pressure (NCPAP) for preterm infants. Cochrane Database Syst. Rev..

[77] Tana M, Tirone C, Aurilia C, Lio A, Paladini A, Fattore S (2023). Respiratory management of the preterm infant: supporting evidence-based practice at the bedside. Children (Basel).

[78] Doyle LW, Cheong JL, Hay S, Manley BJ, Halliday HL (2021). Late (≥ 7 days) systemic postnatal corticosteroids for prevention of bronchopulmonary dysplasia in preterm infants. Cochrane Database Syst. Rev..

[79] Embleton ND, van den Akker CHP, Johnson M (2025). Parenteral nutrition for preterm infants: benefits and risks in 2025. Semin. Fetal Neonatal. Med..

[80] Premji SS, Chessell L (2011). Continuous nasogastric milk feeding versus intermittent bolus milk feeding for premature infants less than 1500 grams. Cochrane Database Syst. Rev..

[81] Ballard O, Morrow AL (2013). Human milk composition: nutrients and bioactive factors. Pediatr. Clin. North Am..

[82] Cantey JB (2016). Optimizing the use of antibacterial agents in the neonatal period. Paediatr. Drugs.

[83] Korpela K, Blakstad EW, Moltu SJ, Strømmen K, Nakstad B, Rønnestad AE (2018). Intestinal microbiota development and gestational age in preterm neonates. Sci. Rep..

[84] Li F, Hooi SL, Choo YM, Teh CSJ, Toh KY, Lim LWZ (2025). Progression of gut microbiome in preterm infants during the first three months. Sci. Rep..

[85] Shen W, Qiu W, Liu Y, Liao W, Ma Y, He Y (2021). Postnatal age is strongly correlated with the early development of the gut microbiome in preterm infants. Transl. Pediatr..

[86] Khan A, Mi H, Gao F, Hu Q, Gu X, Ma F (2023). Dynamic changes of the gut microbial colonization in preterm infants with different time points after birth. Front. Microbiol..

[87] Arboleya S, Binetti A, Salazar N, Fernández N, Solís G, Hernández-Barranco A (2012). Establishment and development of intestinal microbiota in preterm neonates. FEMS Microbiol. Ecol..

[88] Aujoulat F, Roudière L, Picaud JC, Jacquot A, Filleron A, Neveu D (2014). Temporal dynamics of the very premature infant gut dominant microbiota. BMC Microbiol..

[89] Heida FH, Kooi EMW, Wagner J, Nguyen T-Y, Hulscher JBF, van Zoonen AGJF (2021). Weight shapes the intestinal microbiome in preterm infants: results of a prospective observational study. BMC Microbiol..

[90] Stewart CJ, Embleton ND, Marrs EC, Smith DP, Nelson A, Abdulkadir B (2016). Temporal bacterial and metabolic development of the preterm gut reveals specific signatures in health and disease. Microbiome.

[91] Aguilar-Lopez M, Dinsmoor AM, Ho TTB, Donovan SM (2021). A systematic review of the factors influencing microbial colonization of the preterm infant gut. Gut Microbes.

[92] Suárez-Martínez C, Santaella-Pascual M, Yagüe-Guirao G, Martínez-Graci C (2023). Infant gut microbiota colonization: influence of prenatal and postnatal factors, focusing on diet. Front. Microbiol..

[93] Zeng S, Ying J, Li S, Qu Y, Mu D, Wang S (2022). First 1000 days and beyond after birth: gut microbiota and necrotizing enterocolitis in preterm infants. Front. Microbiol..

[94] Tauchi H, Yahagi K, Yamauchi T, Hara T, Yamaoka R, Tsukuda N (2019). Gut microbiota development of preterm infants hospitalised in intensive care units. Benef. Microbes.

[95] Pamela T, Daniel G (2024). How human milk shapes the gut microbiota in preterm infants: potential for optimizing early-life microbial development. Microbiome Res. Rep..

[96] Cong X, Xu W, Janton S, Henderson WA, Matson A, McGrath JM (2016). Gut microbiome developmental patterns in early life of preterm infants: impacts of feeding and gender. PLoS One.

[97] Senn V, Bassler D, Choudhury R, Scholkmann F, Righini-Grunder F, Vuille-dit-Bille RN (2020). Microbial colonization from the fetus to early childhood-a comprehensive review. Front. Cell. Infect. Microbiol..

[98] Chen X, Shi Y (2023). Determinants of microbial colonization in the premature gut. Mol. Med..

[99] Dahl C, Stanislawski M, Iszatt N, Mandal S, Lozupone C, Clemente JC (2017). Gut microbiome of mothers delivering prematurely shows reduced diversity and lower relative abundance of *Bifidobacterium* and *Streptococcus*. PLoS One.

[100] Kamal S, Andersen A, Krych L, Lauridsen C, Sangild P, Thymann T (2019). Preterm birth has effects on gut colonization in piglets within the first 4 weeks of life. J. Pediatr. Gastroenterol. Nutr..

[101] Pan X, Thymann T, Gao F, Sangild P (2020). Rapid Gut adaptation to preterm birth involves feeding-related DNA methylation reprogramming of intestinal genes in pigs. Front. Immunol..

[102] Henderickx JGE, Zwittink RD, van Lingen RA, Knol J, Belzer C (2019). The preterm gut microbiota: an inconspicuous challenge in nutritional neonatal care. Front. Cell. Infect. Microbiol..

[103] Chu DM, Ma J, Prince AL, Antony KM, Seferovic MD, Aagaard KM (2017). Maturation of the infant microbiome community structure and function across multiple body sites and in relation to mode of delivery. Nat. Med..

[104] Chernikova DA, Madan JC, Housman ML, Zain-ul-abideen M, Lundgren SN, Morrison HG (2018). The premature infant gut microbiome during the first 6 weeks of life differs based on gestational maturity at birth. Pediatr. Res..

[105] Lu J, Claud EC (2019). Connection between gut microbiome and brain development in preterm infants. Dev. Psychobiol..

[106] Devarajalu P, Kumar J, Dutta S, Attri SV, Kabeerdoss J (2025). Gut microbiota alteration in healthy preterm infants: an observational study from tertiary care center in India. Microorganisms.

[107] Preidis GA, Versalovic J (2009). Targeting the human microbiome with antibiotics, probiotics, and prebiotics: gastroenterology enters the metagenomics era. Gastroenterology.

[108] Good M, Sodhi CP, Ozolek JA, Buck RH, Goehring KC, Thomas DL (2014). *Lactobacillus rhamnosus* HN001 decreases the severity of necrotizing enterocolitis in neonatal mice and preterm piglets: evidence in mice for a role of TLR9. Am. J. Physiol. Gastrointest Liver Physiol..

[109] Blackwood BP, Yuan CY, Wood DR, Nicolas JD, Grothaus JS, Hunter CJ (2017). Probiotic *Lactobacillus* species strengthen intestinal barrier function and tight junction integrity in experimental necrotizing enterocolitis. J. Probiotics Health.

[110] Hagen PC, Skelley JW (2019). Efficacy of bifidobacterium species in prevention of necrotizing enterocolitis in very-low birth weight infants. a systematic review. J. Pediatr. Pharmacol. Ther..

[111] Oshiro T, Nagata S, Wang C, Takahashi T, Tsuji H, Asahara T (2019). *Bifidobacterium* supplementation of colostrum and breast milk enhances weight gain and metabolic responses associated with microbiota establishment in very-preterm infants. Biomed. Hub.

[112] Ananthan A, Balasubramanian H, Rath C, Muthusamy S, Rao S, Patole S (2024). *Lactobacillus rhamnosus* GG as a probiotic for preterm infants: a strain specific systematic review and meta-analysis. Eur. J. Clin. Nutr..

[113] Zhou KZ, Wu K, Deng LX, Hu M, Luo YX, Zhang LY (2023). Probiotics to prevent necrotizing enterocolitis in very low birth weight infants: a network meta-analysis. Front. Pediatr..

[114] Kulkarni T, Majarikar S, Deshmukh M, Ananthan A, Balasubramanian H, Keil A (2022). Probiotic sepsis in preterm neonatesa systematic review. Eur. J. Pediatr..

[115] Pammi M, Cope J, Tarr PI, Warner BB, Morrow AL, Mai V (2017). Intestinal dysbiosis in preterm infants preceding necrotizing enterocolitis: a systematic review and meta-analysis. Microbiome.

[116] Hou K, Wu Z-X, Chen X-Y, Wang J-Q, Zhang D, Xiao C (2022). Microbiota in health and diseases. Signal Transduct. Target. Ther..

[117] Wang HB, Wang PY, Wang X, Wan YL, Liu YC (2012). Butyrate enhances intestinal epithelial barrier function via up-regulation of tight junction protein Claudin-1 transcription. Dig. Dis. Sci..

[118] Parada Venegas D, De la Fuente MK, Landskron G, González MJ, Quera R, Dijkstra G (2019). Short Chain Fatty Acids (SCFAs)-mediated gut epithelial and immune regulation and its relevance for inflammatory bowel diseases. Front. Immunol..

[119] Bui TNY, Paul A, Guleria S, O'Sullivan JM, Toldi G. 2025. Short-chain fatty acids-a key link between the gut microbiome and Tlymphocytes in neonates? *Pediatr. Res.* https://doi.org/10.1038/s41390-025-04075-0. 10.1038/s41390-025-04075-0 40307498 PMC13221302

[120] Kadivnik M, Plečko D, Kralik K, Arvaj N, Wagner J (2024). Role of IL-6, IL-10 and TNFα gene variants in preterm birth. J. Clin. Med..

[121] Kim J, Park H, Park NY, Hwang SI, Kim YE, Sung SI (2025). Functional maturation of preterm intestinal epithelium through CFTR activation. Commun. Biol..

[122] Wallaeys C, Garcia-Gonzalez N, Libert C (2023). Paneth cells as the cornerstones of intestinal and organismal health: a primer. EMBO Mol. Med..

[123] Heida FH, Beyduz G, Bulthuis MLC, Kooi EMW, Bos AF, Timmer A (2016). Paneth cells in the developing gut: when do they arise and when are they immune competent?. Pediatr. Res..

[124] Schaart MW, de Bruijn ACJM, Schierbeek H, Tibboel D, Renes IB, van Goudoever JB (2009). Small intestinal MUC2 synthesis in human preterm infants. Am. J. Physiol. Gastrointest. Liver Physiol..

[125] Pelaseyed T, Bergström JH, Gustafsson JK, Ermund A, Birchenough GM, Schütte A (2014). The mucus and mucins of the goblet cells and enterocytes provide the first defense line of the gastrointestinal tract and interact with the immune system. Immunol. Rev..

[126] Brown JA, Bashir H, Zeng MY (2025). Lifelong partners: gut microbiota-immune cell interactions from infancy to old age. Mucosal Immunol..

[127] Khailova L, Dvorak K, Arganbright KM, Halpern MD, Kinouchi T, Yajima M (2009). *Bifidobacterium bifidum* improves intestinal integrity in a rat model of necrotizing enterocolitis. Am. J. Physiol. Gastrointest. Liver Physiol..

[128] Mazziotta C, Tognon M, Martini F, Torreggiani E, Rotondo JC (2023). Probiotics mechanism of action on immune cells and beneficial effects on human health. Cells.

[129] Sornplang P, Piyadeatsoontorn S (2016). Probiotic isolates from unconventional sources: a review. J. Anim. Sci. Technol..

[130] Kang M, Yun B, Mun D, Kim S, Jeong KC, Kim Y (2025). Bovine colostrum-derived exosomes alleviate muscle degeneration by modulating gut microbiota and metabolic homeostasis in atrophy models. J. Anim. Sci. Technol..

[131] Patel RM, Denning PW (2015). Intestinal microbiota and its relationship with necrotizing enterocolitis. Pediatr. Res..

[132] Sodhi CP, Neal MD, Siggers R, Sho S, Ma C, Branca MF (2012). Intestinal epithelial Toll-like receptor 4 regulates goblet cell development and is required for necrotizing enterocolitis in mice. Gastroenterology.

[133] Wang J, Gao Y, Cheng C, Li Y, Zhang X, Yao D, *et al*. 2024. Dangguibuxue decoction protects against lipopolysaccharides-induced mastitis in bovine mammary epithelial cells *in Vitro*. *J. Anim. Sci. Technol.* DOI:10.5187/jast.2024.e63. 10.5187/jast.2024.e63 PMC13260098 42291113

[134] Yazji I, Sodhi CP, Lee EK, Good M, Egan CE, Afrazi A (2013). Endothelial TLR4 activation impairs intestinal microcirculatory perfusion in necrotizing enterocolitis via eNOS-NO-nitrite signaling. Proc. Natl. Acad. Sci. USA.

[135] Morrow AL, Lagomarcino AJ, Schibler KR, Taft DH, Yu Z, Wang B (2013). Early microbial and metabolomic signatures predict later onset of necrotizing enterocolitis in preterm infants. Microbiome.

[136] Wang Y, Hoenig JD, Malin KJ, Qamar S, Petrof EO, Sun J (2009). 16S rRNA gene-based analysis of fecal microbiota from preterm infants with and without necrotizing enterocolitis. ISME J..

[137] Schwartz D (2022). #20 Bloodstream infection is associated with prior gut microbiome colonization of the same strain in the neonatal intensive care unit. J. Pediatr. Infect. Dis. Soc..

[138] Gritz EC, Bhandari V (2015). The human neonatal gut microbiome: a brief review. Front. Pediatr..

[139] Beghetti I, Barone M, Brigidi P, Sansavini A, Corvaglia L, Aceti A (2023). Early-life gut microbiota and neurodevelopment in preterm infants: a narrative review. Front. Nutr..

[140] Lu J, Lu L, Yu Y, Oliphant K, Drobyshevsky A, Claud EC (2022). Early preterm infant microbiome impacts adult learning. Sci. Rep..

[141] Carlson AL, Xia K, Azcarate-Peril MA, Goldman BD, Ahn M, Styner MA (2018). Infant gut microbiome associated with cognitive development. Biol. Psychiatry.

[142] Xia J, Claud EC (2023). Gut microbiome-brain axis as an explanation for the risk of poor neurodevelopment outcome in preterm infants with necrotizing enterocolitis. Microorganisms.

[143] Yu Y, Lu L, Sun J, Petrof EO, Claud EC (2016). Preterm infant gut microbiota affects intestinal epithelial development in a humanized microbiome gnotobiotic mouse model. Am. J. Physiol. Gastrointest. Liver Physiol..

[144] Lu J, Lu L, Yu Y, Cluette-Brown J, Martin CR, Claud EC (2018). Effects of intestinal microbiota on brain development in humanized gnotobiotic mice. Sci. Rep..

[145] Wang Y, Cheng T, Cui Y, Qu D, Peng X, Yang L (2024). Associations between gut microbiota and adverse neurodevelopmental outcomes in preterm infants: a two-sample Mendelian randomization study. Front. Neurosci..

[146] Mhanna A, Martini N, Hmaydoosh G, Hamwi G, Jarjanazi M, Zaifah G (2024). The correlation between gut microbiota and both neurotransmitters and mental disorders: a narrative review. Medicine (Baltimore).

[147] Wang Y, Zhu J, Zou N, Zhang L, Wang Y, Zhang M (2023). Pathogenesis from the microbial-gut-brain axis in white matter injury in preterm infants: a review. Front. Integr. Neurosci..

[148] Sherman MP, Zaghouani H, Niklas V (2015). Gut microbiota, the immune system, and diet influence the neonatal gut-brain axis. Pediatr. Res..

[149] Krupa-Kotara K, Gwioździk W, Nandzik S, Grajek M (2023). The role of microbiota pattern in anxiety and stress disorders-a review of the state of knowledge. Psych..

[150] Pärtty A, Kalliomäki M, Wacklin P, Salminen S, Isolauri E (2015). A possible link between early probiotic intervention and the risk of neuropsychiatric disorders later in childhood: a randomized trial. Pediatr. Res..

[151] Bran-Popescu CA, Saulea O, Susanu M, Tropcin E, Vasiliu B, Hofman C, *et al*. 2024. Neurodevelopmental outcome in preterm infants. The Newborn Research & Reviews.

[152] Pinto F, Fernandes E, Virella D, Abrantes A, Neto M (2019). Born preterm: a public health issue. Portuguese J. Public Health.

[153] Van Belkum M, Mendoza Alvarez L, Neu J (2020). Preterm neonatal immunology at the intestinal interface. Cell Mol. Life Sci..

[154] Anderson C, Cacola P (2017). Implications of preterm birth for maternal mental health and infant development. MCN Am. J. Matern. Child Nurs..

